# Psychological and social aspects in orthopaedics and trauma surgery, challenges and solutions in trauma: a didactic overview

**DOI:** 10.1530/EOR-2025-0054

**Published:** 2025-06-02

**Authors:** Josef Grab

**Affiliations:** Insurance Medicine, Suva, Luzern, Switzerland

**Keywords:** stress disorders, post-traumatic, social workers, orthopaedic surgeons, psychological first aid, stereotyping

## Abstract

- **Psychological consequences of trauma**: Acute stress reactions and post-traumatic stress disorder are common psychological conditions that affect the healing process.
- **Early interventions**: Psychological first aid and psychoeducation are evidence-based approaches aimed at mitigating post-traumatic symptoms.
- **Social support**: It plays a central role in psychological stabilisation and promotion of functional recovery.
- **Multidisciplinary approaches**: Cooperation between orthopaedists, psychologists and social workers is crucial for optimal treatment results.
- **Challenges in clinical practice**: Limited time, stigmatisation of mental illness and inadequate resources are common barriers to effective care.

## Introduction

Orthopaedics and trauma surgery are medical specialities that mainly deal with the treatment of injuries and musculoskeletal diseases. Orthopaedic surgeons and trauma specialists often focus on the physical aspects of treatment, whereas rehabilitation adopts a more comprehensive approach by considering the whole person. This holistic perspective is supported by the World Health Organisation’s (WHO) International Classification of Functioning, Disability and Health, which emphasises upon the biopsychosocial aspects of health and illness (1, 2).

The WHO defines health as a state of optimal well-being that goes beyond the mere absence of disease. Moreover, disease is a disruption of normal physical or psychological functions that negatively affects well-being (3). In this context, psychological and social factors play a decisive role, particularly in the treatment of trauma complications.

Enhancing patients’ quality of life is a key focus in the treatment and rehabilitation of patients who experienced trauma. Quality of life encompasses physical, psychological, social and economic aspects of health. Accidents can significantly affect the quality of life by causing long-term health issues, disability and social limitations (4). Therefore, treatment and rehabilitation should focus on restoring physical functionality and supporting the psychological and social reintegration of those affected.

The inclusion of affected individuals in their familiar social environment is crucial in these situations. A comprehensive treatment approach should guarantee that all people, regardless of their abilities or disabilities, can access appropriate support services. This means designing the environment and services that consider and address the needs of all those affected. By fostering inclusion, holistic treatment can improve physical and mental health and support the social participation and integration of those affected into society.

Orthopaedics and trauma surgery face challenges in addressing the physical, psychological and social aspects of trauma treatment. A comprehensive understanding of patient needs is crucial for successful rehabilitation and improvement in the quality of life. This review examines the psychological and social aspects of orthopaedics and trauma surgery, particularly on the treatment of trauma-related consequences. It aims to illustrate how a holistic approach can support healing and reintegration, ultimately improving the quality of life and promoting inclusion. Initially, the psychological consequences of trauma will be discussed. Then, psychological intervention alternatives will be presented before discussing the practical challenges and strategies for improving psychosocial care. Finally, recommended solutions are presented.

## Psychological consequences of trauma

Traumatic events can significantly affect the psychological state of individuals involved. These range from acute psychological reactions to post-traumatic stress disorders (PTSDs). Affected individuals are suddenly displaced from their familiar routines, which can result in considerable social consequences. These subtle factors sometimes influence somatic treatment. This first section provides an overview of the psychological reactions.

### Acute psychological reactions

Acute psychological reactions following traumatic events, including musculoskeletal trauma, can manifest in various ways. Acute psychological reactions must be identified promptly and considered part of the acute care plan. Acute psychological reactions must be considered when planning physical (somatic) treatment.

The acute stress reaction (F43.0 ICD-10) is a temporary reaction to extreme psychological or physical stress such as accidents or experiences of violence. It occurs in individuals with no other apparent mental disorders and subsides within hours or a few days of such events (5). Acute stress disorder (ASD) must be distinguished from an acute stress reaction. ASD usually occurs within 30 days of the event and can include symptoms such as re-experiencing, hyper arousal, numbness, depersonalisation, derealisation, anxiety and avoidance behaviour (6, 7). A study conducted in 2022 examined its prevalence in patients who sustained trauma. The prevalence rate of ASD at baseline was approximately 21.7%, and 36.1% of patients who experienced trauma had PTSD 12 months after the injury. ASD is a risk factor of PTSD development (8). The risk of developing ASD also depends on the trauma type. The results showed that the ASD prevalence ranged from 14.1% in war-related trauma to 36.0% in interpersonal trauma. Interpersonal trauma was significantly more likely to result in ASD in other types of events, except disasters (9).

### Persistent psychological reactions

PTSD (F41.1 ICD-10) may develop if the ASD symptoms persist for longer than 1 month. PTSD is characterised by intrusions (e.g. flashbacks), avoidance behaviour, negative alterations in thinking and emotions and hyperarousal symptoms (5). Many affected individuals can process traumatic experiences relatively well and integrate them into their existing self-concept and worldview. Nevertheless, long-term psychological consequences manifest in a significant proportion of patients with polytrauma. A study of 337 patients with polytrauma who were examined >20 years after the traumatic event revealed psychiatric sequelae in 51.3%. A total of 48.2% and 4.1% of the patients experienced depression and anxiety disorders, respectively (10). Another study showed a PTSD prevalence of 22.6% in patients with polytrauma (11). Despite the high prevalence of psychological issues, many patients also demonstrate positive developments. Approximately one-third of patients reported significant improvements in interpersonal relationships (29.2%), life satisfaction (36.2%) and openness to new opportunities (32.5%) (12). Risk factors for the development of post-traumatic psychological disorders include prior psychiatric treatment, additional psychological stressors after the traumatic event, high levels of neuroticism and negative self-perceptions (10, 11). Studies have shown that long-term psychological problems manifest in a significant proportion of patients with polytrauma. These findings highlight the importance of a comprehensive, long-term approach to the care of patients with polytrauma, addressing psychological and physical aspects. Social support is also crucial in reducing the risk of PTSD and other psychological disorders. Social support can account for up to 40% of the variability in PTSD symptoms (13, 14). Social support facilitates recovery in various mechanisms. It reduces psychological stress and improves coping mechanisms. An increase in self-esteem is one of the outcomes of such support. In addition, it fosters a sense of belonging. As a result, patients are better able to utilise existing coping strategies or even develop new ones. This quickly alleviates PTSD symptoms and lowers the recurrence risk (15).

Psychological symptoms may arise in individuals who have experienced less severe traumatic experience and those with polytrauma. Even minor trauma can have psychological effects. The symptomatology and severity of these effects depend on individual and social factors. Studies have emphasised the importance of early intervention and social support in preventing long-term negative consequences (16, 17, 18, 19).

## Psychological interventions in trauma surgery

If secondary psychological consequences arise following traumatic events, orthopaedic–trauma surgical treatment must be adjusted accordingly. Interventions must be tailored to the treatment phase. The following section provides an overview of the relevant concepts.

### Acute phase

Five aspects of psychological interventions are described in the acute phase, which will now be examined in greater detail. The goal is to ensure safety and orientation, achieve emotional stabilisation, activate resources, promote social support and implement psychoeducation.

Psychological first aid is an evidence-based approach designed to reduce stress and promote adaptive functioning immediately following traumatic events. It focuses on enhancing immediate and sustained safety, providing physical and emotional comfort and offering reassurance and orientation to emotionally overwhelmed individuals (20).

Early psychological interventions aim to stabilise acute post-traumatic symptoms. They intend to normalise early post-traumatic reactions. Coping strategies for post-traumatic symptoms are taught to patients, along with relaxation techniques and stress reduction methods (21, 22). In 2023, a study on trauma-informed care in psychiatric wards also underlines the importance of the stabilisation phase. The authors stress that stabilisation is considered primary and essential for overcoming trauma-related difficulties (23).

Psychological treatments focus on activating and strengthening existing coping mechanisms of patients. Such treatments aim to promote problem-solving skills and regulating emotional skills. In addition, adaptive coping strategies are improved (24, 25).

As previously discussed, social support is a crucial pillar, indicating the importance of the rapid initiation of social support. Implementing supportive measures when establishing contact with primary caregivers may be necessary. Community resources should also be mobilised. Family members can be involved in the interventions (26).

A key element of psychoeducation is providing information on stress reactions and coping mechanisms. Affected individuals should be informed about the most common post-traumatic reactions. Moreover, the distinction between adaptive and maladaptive coping should be explained to them. Finally, individuals should be made aware of when to seek additional help (27).

The ‘Trauma Toolkit’ emphasises the importance of safety as one of the core principles of trauma-informed care. It emphasises that all clients should have individualised safety plans that are fully integrated into programme activities (28). The central role of safety and stabilisation in the early phase of trauma treatment is discussed based on existing research evidence. Trauma-informative interventions should be seen as a complex and multilayered framework that currently lacks an overarching structure and clear theoretical foundations. These factors complicate the summarisation of findings, which should be tailored to specific environments and target groups (29, 30).

In summary, the indication for psychological interventions relevant even in the early phase. Prompt treatment mitigates secondary effects and provides a strong foundation for the subsequent rehabilitation phase.

### Rehabilitation phase

The effectiveness of the treatment and successful restoration of functionality largely depend on the quality of the subsequent rehabilitation phase. Inadequate rehabilitative care following the acute phase must be avoided because it significantly reduces the effectiveness of treatment for patients with acute trauma (31). Rehabilitation following severe accidents should be distinguished from regular orthopaedic rehabilitation after elective surgeries. The rehabilitative approach following severe accidents is divided into six phases (32, 33). To ensure effective coordination of rehabilitative measures, trauma rehabilitation centres should be integrated into current trauma networks. This integration is anticipated to improve patient outcomes (34). The initiation of early rehabilitation in the intensive care unit is underconsidered, as early intervention may accelerate recovery and yield improved functional outcomes (35, 36, 37).

Psychosocial factors are crucial in rehabilitation. Stress, depression and anxiety can hinder rehabilitation and result in suboptimal outcomes. Early detection and intervention to address psychological stress are crucial in optimising the effectiveness of rehabilitation (38). Psychosocial support in early rehabilitation can positively affect the quality of life (39). In addition, social support from family and friends may positively influence the healing process by reducing patients’ anxiety and guaranteeing security (15).

In 2024, a study examined the long-term recovery of patients following orthopaedic trauma over 18 months (40). The results indicate that recovery is a complex process influenced by various factors, such as the severity of the injury and available social support. Patients who have experienced accidents and trauma often suffer from chronic issues such as pain, anxiety, depression and limited mobility (40, 41). Patients who have traumatic experiences often report chronic health issues that are not adequately addressed by the healthcare system (40).

### Return to the workplace

All therapeutic interventions, in both acute treatment and rehabilitation, are intended to ensure the professional reintegration of patients of working age. The severity of the injury is a key factor in returning to work (42). Studies have indicated that severe pain can delay return to work. Patients with higher pain perception are less likely to return to work within a given time frame than those with lower pain perception (42, 43). Beyond physical injuries, returning to work can be facilitated or hindered by psychosocial factors. High self-efficacy levels are a significant predictor of rapid reintegration into working life. Patients who consider themselves competent in managing their health have a higher success rate in returning to work (42). Patients’ attitudes regarding their expectations for recovery are also significant. Those with an optimistic outlook are more likely to return to work earlier (42, 44). The psychological state of the patients, including their expectations, can significantly influence rehabilitation outcomes. Generally, patients who have positive expectations and possess a strong sense of self-efficacy achieve more favourable recovery outcomes (45). Social status and educational level can facilitate returning to work, as higher social status and education levels are often associated with better job market opportunities, resulting in a shift from physically demanding tasks to less strenuous ones (42). In addition to somatic and psychosocial aspects, socioeconomic factors must be considered when returning to work, as compensation status and legal disputes can affect workplace reintegration (42, 43).

In summary, returning to work depends on various biopsychosocial factors. A comprehensive consideration of these factors can contribute to optimising the overall treatment and facilitating return to work.

## Challenges in orthopaedic trauma surgery practice

The previous chapter focuses on psychological and social factors following accidents and their influence on treatment. The resulting challenges for orthopaedic trauma surgery practice are outlined below with a discussion on appropriate strategies for overcoming them.

The scientific literature highlights the broad spectrum of challenges in orthopaedic and trauma surgery. The psychosocial aspects that also play a significant role in this context are discussed in detail. Approximately 50% of patients with orthopaedic injuries experience psychosocial stress. This may manifest as depression, anxiety or post-traumatic stress (46). These psychological burdens can negatively affect the healing process, intensify chronic pain and restrict mobility (46, 47). Extra care must be taken when performing surgical interventions on patients with ASD. The accompanying psychological instability may disrupt the healing process (48, 49). Postoperative satisfaction and patient-reported outcomes are influenced by patient expectations. Positive expectations usually correlate with better results and reduced pain (50).

Orthopaedic and trauma surgery professionals encounter various challenges, including time constraints, stigma surrounding mental illness and a lack of knowledge about available psychosocial services (46, 51). Despite the growing scientific consensus on the positive influence of psychosocial support on the healing process, the use of structured care models is inadequately implemented in many orthopaedic institutions (51, 52). In practice, collaboration between multidisciplinary teams can enhance the quality of care, minimise morbidity and reduce mortality (53, 54). Multidisciplinary teams enable a holistic view of the patient’s needs and tailored targeted treatment, whereby cooperation between different specialists is crucial, particularly in complex injuries, to ensure optimal treatment outcomes. In this regard, close and effective collaboration between orthopaedic surgeons, traumatologists, physiotherapists and other relevant specialists is essential (53, 55). Therefore, pursuing an integrated therapy approach is advisable, particularly when caring for patients with complex traumatic experiences. Patients with untreated psychosocial stress have significantly poorer outcomes in terms of quality of life, functionality and treatment satisfaction (46, 56, 57). Psychological interventions, such as cognitive behavioural therapy, may alleviate symptoms such as depression and anxiety and improve the rehabilitation process (46). Psychological factors, such as depression and social support, are recognised as significant predictors of surgical outcomes (58). The provision of psychological care is clinically effective and cost-efficient. Over time, it can lead to a decrease in healthcare costs (51).

The development of an integrated therapeutic approach requires minimising diagnostic challenges. Initially, an accurate somatic diagnosis must be made. Moreover, the necessity of standardised protocols for imaging and diagnosis is emphasised (59). In patients with multiple traumas, minor injuries may be overlooked, as the focus is often on life-threatening injuries. This necessitates a systematic examination of all body parts (59). In addition, psychological stress, such as anxiety, depression or post-traumatic stress, can increase the perception of pain and discomfort. This can result in discrepancies between objective findings and subjective symptoms (60, 61, 62, 63, 64, 65, 66). Moreover, orthopaedic professionals often show reservations regarding psychosocial factors (67). Patients with similar injuries may present different complaints and treatment goals due to varying psychosocial circumstances (57). Unfortunately, no uniform criteria have been established for orthopaedic diagnoses, which results in inconsistencies. For example, the assessment of pain and functionality between different clinics and specialists vary considerably (68).

Healthcare institutions experience complex financial and structural challenges, which affect the extent of patient care. Reimbursement rates for therapies are continuously being reduced; thus, efficient billing strategies and alternative revenue sources must be developed to maintain profitability (69, 70). The financial burden on patients should not be ignored. Many patients have financial worries, particularly if they are unable to work (71, 72, 73).

Psychosocial factors, multidisciplinary approaches and financial considerations are crucial in determining the indications for trauma surgery. Integrating these factors may improve patient care and outcomes. These insights significantly influence decision-making in trauma surgery. Despite the associated difficulties, they should be considered when determining the indication.

## Strategies for improving psychosocial care

The overriding aim of medical practice is to provide patients with the best possible treatment. Based on the findings of the challenges in orthopaedic practice related to trauma surgery, the focus is on the development of strategies for better integration of psychosocial care into the treatment plan.

Leadership support in implementing an integrated care model is critical to the successful integration of mental health services. This includes providing resources and promoting a culture that values psychosocial care. Psychosocial care must be culturally embedded as an integral part of patient care (46, 51, 74, 75). Evidence-based training in psychosocial care within orthopaedic facilities is crucial to optimise healthcare providers’ understanding and competencies in this field. Training should aim to provide a fundamental understanding of the psychosocial factors that affect the recovery of patients with orthopaedic problems and traumatic experiences. This includes the relevance of depression, anxiety and other psychological burdens for treatment outcomes (74). Furthermore, training can help reduce stigmatisation by fostering a deeper understanding of mental health and highlighting the relevance of psychosocial care (46, 67, 76). In summary, the implementation of psychosocial services in orthopaedic facilities may significantly reduce healthcare costs over time. This development is attributed to various factors, such as improved treatment outcomes, reduced hospital stays and decreased necessity for follow-up treatments (46, 77, 78).

## Conclusion

The integration of psychological and social aspects into orthopaedic–trauma treatment is crucial for achieving sustainable improvements in patients’ quality of life. The physical and profound psychological and social effects of traumatic events can significantly influence the healing process. Long-term recovery success is achieved through holistic rehabilitation that adequately takes into account the physical, psychological and social dimensions.

Early interventions, such as psychoeducation, social support and psychological care (e.g. cognitive behavioural therapy), can significantly reduce the risk of PTSDs and other psychological sequelae (65, 79, 80). Social inclusion, understood as reintegration into the social environment, plays a crucial role in rehabilitation. Social support contributes to both psychological stabilisation and promotion of functional recovery (81, 82, 83). The achievement of the aforementioned treatment goals can be ensured through multidisciplinary teams. Cooperation between orthopaedic surgeons, psychologists and rehabilitation specialists enables the provision of comprehensive care that addresses both physical and psychosocial requirements (46). Considering biopsychosocial factors can significantly improve the long-term patient outlook following optimised treatment. This development manifests in an increased number of immediate treatment outcomes. Moreover, a positive influence on the patient’s long-term quality of life is also evident (46).

Optimising patient care requires early psychological support. This can be achieved through training the medical staff to recognise psychological stress and integrating psychologists into orthopaedic teams. In addition, doctor–patient communication must be improved. Better communication results in greater patient satisfaction. Regular communication training sessions for medical staff and the implementation of feedback systems for patients are desirable. Moreover, social support networks contribute to the promotion of the social integration of patients with surgical trauma. Therefore, patients should be directed to self-help groups and online platforms. Interdisciplinary approaches also promote collaboration among orthopaedic surgeons, psychologists and social workers. Regular interdisciplinary case discussions foster mutual understanding and contribute to the optimisation of treatment planning. Finally, raising awareness among medical staff about the psychosomatic aspects of trauma surgery is crucial, which can be achieved through appropriate training.

Apart from the implementation of the aforementioned improvement measures, future research should focus on the development of standardised protocols for the integration of psychosocial care into orthopaedic treatment plans and evaluate their effectiveness in various contexts. The use of innovative approaches, such as trauma-informed care or digital support services, could be promising in this context.

Integrating psychosocial factors into orthopaedic and trauma care presents an opportunity to significantly improve patients’ quality of life, treatment outcomes and long-term recovery. Orthopaedic surgeons and trauma specialists can significantly contribute to the advancement of holistic patient care by incorporating these recommendations, thereby shaping the future of orthopaedic and trauma medicine in the United States.

## ICMJE Statement of Interest

The author declares that there is no conflict of interest that could be perceived as prejudicing the impartiality of the work reported.

## Funding Statement

This work did not receive any specific grant from any funding agency in the public, commercial or not-for-profit sector.
